# Fangjihuangqi Decoction inhibits MDA‐MB‐231 cell invasion in vitro and decreases tumor growth and metastasis in triple‐negative breast cancer xenografts tumor zebrafish model

**DOI:** 10.1002/cam4.2894

**Published:** 2020-02-09

**Authors:** Yubo Guo, Yingyi Fan, Xiaohua Pei

**Affiliations:** ^1^ Beijing University of Chinese Medicine Third Affiliated Hospital Beijing University of Chinese Medicine Beijing China; ^2^ Beijing University of Chinese Medicine Fangshan Traditional Medical Hospital Beijing University of Chinese Medicine Beijing China

**Keywords:** breast cancer xenograft tumor, epithelial‐mesenchymal transition, fangjihuangqi decoction, triple‐negative breast cancer, zebrafish

## Abstract

Triple‐negative breast cancer (TNBC) is a basal‐like cancer which is considered to be more intrusive, have a poorer prognosis and chemoresistance. TNBC is characterized by the presence of epithelial to mesenchymal transition (EMT) that plays a major role in the progression of the cancer. In the present study, we first use a classic prescription of Chinese medicine Fangjihuangqi Decoction to treat TGFβ1‐induced MDA‐MB‐231 cells in vitro. Our data showed that TGFβ1‐induced MDA‐MB‐231 cell morphology change, promoted MDA‐MB 231 invasion, increased Vimentin expression, and decreased E‐cadherin expression. Further, Fangjihuangqi Decoction‐medicated serum (FHS) treated both MDA‐MB 231 cells and TGFβ1‐induced MDA‐MB‐231 cells. Results showed that Fangjihuangqi Decoction could inhibit cell proliferation, reduce cell invasion, increase E‐cadherin expression, and decrease EMT markers. Secondly, we established a xenograft tumor zebrafish model to assess Fangjihuangqi Decoction inhibition of cancer cell proliferation and invasion. Our results indicated that Fangjihuangqi Decoction could inhibit tumor growth, restrain the sprouts number of tumor neovascularization, and reduce the length of tumor neoplastic lymphatics by increasing E‐cadherin expression and decreasing EMT markers in TNBC xenograft tumor zebrafish model. Overall, our studies provide evidences that Fangjihuangqi Decoction could inhibit TNBC, reverse EMT, and contribute to antimetastasis by increasing E‐cadherin expression and decreasing EMT markers, which provide an experimental basis for clinical application of Fangjihuangqi Decoction on TNBC treatment.

## Graphical abstract

Fangjihuangqi Decoction could inhibit MDA‐MB‐231 cell invasion in vitro and decrease tumor growth and metastasis in triple‐negative breast cancer xenografts tumor model zebrafish

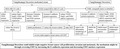

## 1 INTRODUCTION

Breast cancer is the most commonly occurring cancer among women in most countries.^1^ According to global data research in 2018, breast cancer incidence ratio of women is 24.2% and mortality ratio is 15.0%. Furthermore, breast cancer has surpassed lung cancer and ranks first in female cancer deaths.^2^ Although some progress in breast cancer biological and clinical diagnosis and treatment has been made in recent years, it is still the leading cause of death due to cancer in women.^3^ The distant metastasis ratio of breast cancer‐related death is up to 90%.^4^ The death rate due to metastatic breast cancer is nearly the same as 50 years ago.^5^ In 2014, approximately 230 000 women in the United States were diagnosed and 40 000 died of invasive breast cancer.^6^ Triple‐negative breast cancer (TNBC) is a breast cancer with negative immunohistochemical of estrogen receptor (ER), progesterone receptor (PR), and proto‐oncogene Her‐2. Because of its ER (−), PR (−), Her‐2(−), endocrine drugs, such as tamoxifen, for ER (+) and PR (+) breast cancer treatment are ineffective. Targeted drugs, such as Herceptin, for Her‐2 (+) breast cancer treatments are also ineffective. Hormonal therapy, chemotherapy, and radiotherapy are currently used for TNBC treatment, on which TNBC patients often develop anticancer resistance and strong side effects.^7^ TNBC is more aggressive, with local recurrence, organ metastasis (lungs, liver, bones, and brain), and poor prognosis.^8^, ^9^ Besides, TNBC patients had worse overall survival (OS) than patients with non‐TNBC characteristics^10^ and the median OS of patients with metastatic TNBC is approximately 9‐12 months, which leads to an urgent need for new targeted therapies for TNBC and metastatic.^11^

Epithelial‐mesenchymal transition (EMT) is the process of epithelial cells to obtain mesenchymal phenotype. Epithelial cells have tight junction, while mesenchymal cells lack cell junctions and therefore are loose, mobile, and invasive, which are intrinsic properties of mesenchymal cells.^12^ EMT is a prerequisite for tumors to invade and transfer to normal tissues.^13^ Studies have shown that EMT is a complex and key biological process, not only in embryonic development, but also in the metastatic potential of malignant tumors.^14^, ^15^ Breast cancer metastasis begins with local invasion of the surrounding tumor cells by the primary tumor cells and continues until the tumor cells invade and infiltrate into the blood or lymphatic vessels.^16^ Tumor cells spread to the distant organs through the bloodstream or lymphatic vessels, infiltrate the organ parenchyma, proliferate, and promote the formation of angiogenesis and lymphangiogenesis in the organs.^17^ The formation of tumor neovascularization provides important clues for distant metastasis and invasion of the tumor since adequate blood supply is essential for maintaining rapid tumor growth and distant metastasis.^18^ Tumor lymphatic metastasis is formed by tumor cells inducing the formation of primitive and neoplastic lymphatic vessels and invading the surrounding lymphatic vessels in the tumor stroma.^19^ In order to achieve an invasive phenotype, tumor cells need to migrate from a restricted primary site. EMT disrupts cell adhesion by reducing epithelial marker such as E‐cadherin and expressing mesenchymal marker such as Vimentin. After cell adhesion loss, cell polarity changes from apical‐substrate polarity to anterior‐posterior polarity, triggering cell migration and enhancing invasiveness. Epithelial‐mesenchymal transition as a new target of prime interest for anticancer metastasis and chemoresistance in breast cancer therapy has drawn great attention.^20^, ^21^ Studies have shown that EMT biomarkers play a key role in breast cancer metastasis. Families of the zinc‐finger protein Snails (Snail1, Snail2, Snail3), the E‐box‐binding proteins Zebs (Zeb1, Zeb2), the basic helix‐loop‐helix protein Twists (Twist1, Twist2) directly inhibit the expression of E‐cadherin in breast cells by directly binding to E‐box of the proximal promoter of CDH1.^22^, ^23^, ^24^, ^25^ The loss or repositioning of E‐cadherin dissolves the adhesion junction, which is the key to EMT.^26^ What's more, it is reported that TGF‐β1 is a potent EMT inducer, which is elevated in plasma of breast cancer patients and at invasive fronts in human breast cancer tissues, and associated with lymph node metastasis.^27^

Fangjihuangqi Decoction, which is a classic prescription of Chinese medicine, containing *Radix Stephaniae tetrandrae, Radix Astragali*, *Rhizoma Atractylodis macrocephalae*, *Radix glycyrrhizae*, was first recorded in “Jin Gui Yao Lue” by Zhang Zhongjing in Han Dynasty. Fangjihuangqi Decoction has also been clinically used to treat upper extremity edema, fatigue, and enhance immunity to prevent cancer recurrence in postoperative patients with breast cancer.^28^ However, whether Fangjihuangqi Decoction could prevent metastasis or recurrence of breast cancer, the mechanism is still unclear. In this study, we propose to examine the effect of Fangjihuangqi Decoction on triple‐negative breast cancer and EMT in TGFβ1‐treated MDA‐MB‐231 cells and xenograft tumor zebrafish model, which as a chemotherapy drug is clinically more convenient and helpful for TNBC treatment.

## 2 MATERIAL AND METHODS

### 2.1 Preparation of serum containing Fangjihuangqi Decoction (medicated serum)

Female Sprague Dawley rats (body weight, 170 ± 30 g) were purchased from Beijing SiBeiFu Animal Technology Co. Ltd., license number SCXK (Beijing) 2016‐0002. The rats were housed in the clean level condition animal housing facilities (certification number SCXK [Jing] 2011‐0024) of BUCM, temperature of 22°C, humidity of 55%, and a 12‐hour light/dark cycle with free access to tap water and food. The experimental procedures and protocols were reviewed and approved by the Animal Care Committee of BUCM, China.

Fangjihuangqi Decoction granules were composed of 12 g of *Stephania tetrandra S Moore*, 20 g of *Radix Astragali*, 10 g of *Rhizoma Atractylodis Macrocephalae*, and 9 g of *Radix Glycyrrhizae*. The drugs of Fangjihuangqi Decoction were quality controlled according to the Standard Operation Procedure of Chinese Pharmacopoeia and provided by the Chinese pharmacy of Third Affiliated Hospital of Beijing University of Chinese Medicine. Fangjihuangqi Decoction drug‐containing serum was prepared according to the literation.^29^ Fangjihuangqi Decoction granules were dissolved, boiled, and concentrated to a final concentration of 1 g/mL. The SD rats were then randomly divided into two groups: control group (n = 10) and Fangjihuangqi Decoction group (n = 10). Rats were given Fangjihuangqi Decoction granules (36.5 g/kg, five times the amount of the equivalent dose conversion coefficient) by gavage twice daily for consecutively 3d for five times by 1 mL/100 g body weight. The daily intragastric administration dose of rats is 5 times of the equivalent conversion dose of 60 kg adult. Control group was treated with distilled water. All of rats were intraperitoneally anesthetized by 1% pentobarbital sodium after 1 hour for the last administration. Blood was collected from abdominal aorta, centrifuged at 12 000 rpm for 15 minutes, filtered with 0.22‐μm sterile filtration membrane, and stored in a −20°C refrigerator. Fangjihuangqi Decoction‐medicated serum (FHS), which was diluted to the desired concentration with RPMI1640 medium.

### 2.2 Cell culture

Human breast cancer MDA‐MB‐231 cells obtained from Cell Resource Center, Shanghai Institutes for Biological Sciences, Chinese Academy of Sciences were cultured in RPMI Medium 1640 (Gibco) containing 10% fetal bovine serum (FBS, ExCell Bio Inc). Cells were incubated in humidified 95% O_2_ air and 5% CO_2_ atmosphere at 37°C.

### 2.3 Chemicals and antibodies

Rabbit anti‐E‐cadherin Polyclonal antibody (Cat. No:20874‐1‐AP), Mouse anti‐Vimentin Monoclonal antibody (Cat.No:60330‐1‐Ig), Rabbit anti‐TGF‐beta 1 Polyclonal antibody (Cat. No:21898‐1‐AP), Rabbit anti‐ZEB1 Polyclonal antibody (Cat.No:21544‐1‐AP), Rabbit anti‐ZEB2 Polyclonal antibody (Cat. No:14026‐1‐AP), Rabbit anti‐Snail2 Polyclonal antibody (Cat. No:12129‐1‐AP), Rabbit anti‐TWIST1‐specific Polyclonal antibody (Cat. No:25465‐1‐AP), Mouse anti‐beta Actin Monoclonal antibody(66009‐1‐Ig), Recombinant Human TGF beta1(Cat. No: HZ‐1011), Rhodamine (TRITC)‐conjugated Goat Anti‐Mouse IgG (H + L) (Cat. No:SA00007‐1), and Rhodamine (TRITC)‐conjugated Goat Anti‐Rabbit IgG (H + L) (Cat. No:SA00007‐2) were purchased from Proteintech Group, Inc. RPMI Medium 1640 (Gibco, Lot.No:1960297), TRIzol^TM^ Reagent (Invitrogen, Cat. No:15596026 and 15596018), fetal bovine serum (ExCell Bio Inc, Australia, Lot.No:11G047), MatrigelMatrix Basement Membrane 5 millilitres (Corning, Lot. No: 7345017), Whole protein extraction kit (Cat. No: KGP2100, Nanjing Keygen Biotech. Co., Ltd), BCA protein concentration assay kit (Cat. No: KGPBCA, Nanjing Keygen Biotech. Co., Ltd, China), Power SYBRGreen PCR Master Mix (Thermo Fisher Scientific, Lot. No:1804573), DAPI (Solarbio, Cat. No:C0065), RevertAid First Strand cDNA Synthesis Kit (Thermo Fisher Scientific, Lot.No:00638610). Hypersensitive ECL Chemiluminescence Detection Kit (Proteintech, Inc, Lot. No: B2201809), Thalidomide (Cat. No: T0213, Target Molecule Corp.). Cisplatin (Cat. No: K1520124) was purchased from Shanghai Aladdin Biochemical Technology Co., Ltd. Bevacizumab (Cat. No: H0158) was purchased from Shanghai Roche Pharmaceutical Co., Ltd.

### 2.4 Morphological changes in TGFβ1‐treated MDA‐MB‐231 cells

Logarithmic growth phase MDA‐MB‐231 cells were plated at a density of 1 × 10^5^ cells/well in 6‐well plates and the cell morphology was observed under light microscope after administering with TGFβ1 (0, 2.5, 5, 10, 20 ng/mL) for 48 hours.

### 2.5 Cell proliferation assay

MDA‐MB‐231 cells (8 × 10^4^) were seeded in 96‐well plates. Drugs were added to the cells at variable concentrations. At 24, 48, and 72 hours after treatment, 10‐μL 3‐(4,5‐Dimethylthiazol‐2‐yl)‐2,5‐diphenyltetrazolium bromide (MTT) dye solution was added for an additional 4 hours. Subsequently, the cell culture medium was removed and 150 μL/well DMSO was added to dissolve formazan crystals. The optical density value at the wavelength of 490 nm was measured by a FLUOstar Omega Multi‐Function Microplate Reader (BMG Labtech, Germany). IC50 values were calculated using GraphPad Prism 5.01.

### 2.6 Transwell invasion assay

Cell invasion assays were performed using the Corning Transwell Chamber and Matrigel. Matrigel was dissolved at 4°C, followed by a 1:3 dilution with 1640 medium at 37°C to solidify the gel for 1 hour. One percent BSA serum‐free 1640 medium was hydrated for 30 minutes at 37°C. MDA‐MB‐231 cells at a density of 1 × 10^4^ cells/well were trypsinized and suspended in serum‐free RPMI 1640 medium containing different treatment drugs and placed in the upper chambers. RPMI 1640 medium supplemented with 10% fetal bovine serum was placed in the lower chamber. Cells were allowed to invade for 24 hours at 37°C. Invaded cells were fixed with 4% paraformaldehyde for 30 minutes, stained with 0.1% crystal violet for 10 minutes, and counted at magnification ×100 from five fields. The invasive cells degraded the Matrigel matrix layer, migrated, and adhered to the bottom of the polycarbonate membrane. The cell invasive ability was determined by counting the number of cells at the bottom of the polycarbonate membrane with Image Pro Plus 6.0 software. Cisplatin (CDDP, 10 μmol/L) was the positive control.

### 2.7 Immunofluorescence

MDA‐MB‐231 cells at a density of 3 × 10^5^ cells/well were seeded on 35‐mm culture dishes and treated with different dosages of TGFβ1 for 48 hours at 37°C. Immunofluorescence was conducted according to the procedure as previously described with some modifications.^30^ The cells were fixed with 4% paraformaldehyde for 20 minutes and permeabilized by 0.2% Triton X‐100/PBS for 10 minutes at room temperature. Then, 10% goat serum was added to the cells to block for 30 minutes. Primary antibody (TGFβ1, E‐cadherin, Vimentin, 1:50) was detected by incubation with a Rhodamine (TRITC)‐conjugated Goat Anti‐Mouse IgG (H + L) or Rhodamine (TRITC)‐conjugated Goat Anti‐Rabbit IgG (H + L) secondary antibody. DAPI was used to stain the nucleus and was observed at magnification ×600 with laser scanning confocal microscope. Statistical analysis with Image Pro Plus 6.0 software was used.

### 2.8 Zebrafish care and maintenance

Wild‐type AB strain zebrafish embryos were obtained by natural mating. Adult zebrafish were housed in a light‐ and temperature‐controlled aquaculture facility with a standard 14:10 hour light/dark photoperiod and fed with live brine shrimp twice and dry flake once a day. Four to five pairs of zebrafish were set up for nature mating and 200‐300 embryos were generated averagely. Zebrafish embryos of 2dpf were selected, with 30 tails per group. The temperature to maintain is 28.0 ± 0.5°C. Water quality: 200 mg of instant sea salt per 1L of reverse osmosis water, conductivity 480‐510 μS/cm, maintenance temperature 28.0 ± 0.5°C, pH 6.9‐7.2, and hardness 53.7‐71.6 mg/L CaCO_3_. The animal's license number was SYXK (Zhe) 2012‐0171. This research was approved by the international certification of Association for Assessment and Accreditation of Laboratory Animal Care (AAALAC).

To establish human TNBC xenograft tumor in zebrafish model, the TGFβ1‐treated MDA‐MB‐231 cells at a density of 2 × 10^5^ cells/mL were induced with 10 ng/mL TGF‐β1 for 48 hours. Followed by labeling with CM‐DiI, which is a red fluorescent dye well suited for monitoring cell movement or location, 200 cells per tail were microinjected into 2dpf normal wild‐type AB strain or transgenic vascular green fluorescent zebrafish yolk sac.

### 2.9 Growth inhibition of TNBC xenograft tumor and Hematoxylin and eosin (HE) staining

TGFβ1‐treated MDA‐MB‐231 cells were microinjected into 2dpf normal wild‐type AB strain zebrafish yolk sac to establish human TNBC xenograft tumor in zebrafish model. When cultured to 3dpf at 35°C, 30 tails/well were selected under the microscope and randomly assigned to 6‐well plates. Fangjihuangqi Decoction (55.5, 111, and 222 μg/mL) group, the control group, the model group, and cisplatin (CDDP, 15 μg/mL) group were set up. When treated to 5dpf at 35°C, we randomly chose 10 zebrafish under the fluorescence microscope. Image analysis was performed using Image pro plus 6.0 software to calculate the sum of fluorescence intensity of TNBC xenograft tumor to evaluate the tumor growth inhibition ratio of Fangjihuangqi Decoction on TNBC xenograft tumor in zebrafish. Furthermore, the TNBC xenograft tumor zebrafish were fixed in 4% paraformaldehyde at 4°C overnight, dehydrated, cleared, embedded, and sectioned 5 µm for H&E staining. After the tablets were sealed, the changes in tumor cells and tissue structure were observed under a microscope.

### 2.10 Detection of tumor angiogenesis and lymphangiogenesis

TGFβ1‐treated MDA‐MB‐231 cells were transplanted into 2dpf transgenic vascular green fluorescent zebrafish yolk sac by microinjection to establish TNBC xenograft tumor in fluorescent zebrafish model. The normal control group, the model group, the thalidomide group (219 μg/mL), and the FH groups were set and treated to 4dpf at 35°C. The neovascularization buds in the intestine were observed under a laser confocal microscope. In addition, the normal control group, the model group, the bevacizumab group (250 ng/tail), and the FH groups were set and the TNBC xenograft tumor zebrafish were treated to 3dpf at 35°C. Inject rhodamine‐labeled dextran (TRITC‐Dextran) intravenously into each group of zebrafish for angiography under the optical microscope to get rid of vascular interference, and then the lymphatic vessels above the zebrafish cloaca were observed under a laser confocal microscope. The number of buds in the intestine and the total length of lymphatic vessels of three individual segments above the zebrafish cloaca were used to evaluate the inhibition ratio of FH on neovascularization and lymphatic vessels.

### 2.11 Western blotting analysis

Western blotting was conducted according to the procedure as previously described with some modifications.^31^ Whole protein extraction kit (Cat. No: KGP2100, Nanjing Keygen Biotech. Co., Ltd,) and BCA protein concentration assay kit (Cat. No: KGPBCA, Nanjing Keygen Biotech. Co., Ltd) were used for the protein quantification in TGFβ1‐treated MDA‐MB‐231 cells and zebrafish embryos for 48 hours after administration. 25μg cell protein and 80μg zebrafish protein sample were loaded in the 10% sodium dodecyl sulfate‐polyacrylamide gel (SDS‐PAGE) electrophoresis, then transferred into a PVDF membrane, and blocked by 5% skimmed milk. Primary antibody (Rabbit anti‐TGF‐beta 1 Polyclonal antibody, Rabbit anti‐E‐cadherin Polyclonal antibody, Mouse anti‐Vimentin Monoclonal antibody, Rabbit anti‐ZEB1 Polyclonal antibody, Rabbit anti‐ZEB2 Polyclonal antibody, Rabbit anti‐Snail2 Polyclonal antibody, Rabbit anti‐TWIST1‐specific Polyclonal antibody, Mouse anti‐beta Actin Monoclonal antibody, 1:500) was kept overnight at 4°C. Secondary antibody was with horseradish enzyme‐labeled goat anti‐rabbit IgG or horseradish enzyme‐labeled goat anti‐mouse IgG (1:1000). Immunopositive bands were visualized with a hypersensitive ECL chemiluminescence liquid and the images were captured with Azure c500 Bio‐imaging systems. The grayscale values of the blots were quantified using the ImageJ software and normalized with the corresponding β‐actin as the internal control.

### 2.12 RNA extraction and RT‐qPCR

TGFβ1‐treated MDA‐MB‐231 cells and 5dpf zebrafish embryos after administration group of drugs for 48 hours were harvested. We analyzed mRNA levels by RT‐qPCR. Total RNA was extracted using Trizol reagent (Invitrogen). Chloroform separates the sample into a water layer and an organic layer, and RNA is present in the water layer. RNA was precipitated by isopropanol, and washed with 75% ethanol. Then, 50 μL of RNase‐free DEPC water was added to dissolve the RNA. Total RNA was quantified by absorbance at 260/280 nm in a Merinton SMA4000 UV‐Vis Micro‐Spectrophotometer. Two microgram RNA was then used as a template for cDNA synthesis by RevertAid First Strand cDNA Synthesis Kit (Thermo Fisher Scientific) in a Thermo Scientific™ Arktik™ PCR Thermal Cycler. mRNA quantity was determined with the RT‐qPCR in Fluorescence quantitative PCR system (BIO‐RAD), using primer sequences summarized in Table 1 and Power SYBRGreen PCR Master Mix (Thermo Fisher Scientific). The RT‐qPCR cycle was pre‐untwisted for 10min at 95°C, untwisted at 95°C for 15 seconds, annealed at 60°C for 20 seconds, and extended at 72°C for 20 seconds, repeating 40 cycles. The RT‐qPCR results were analyzed by the relative quantitative 2^−△△CT^ method. The experiments were triplicated.

**Table 1:** Primers used in RT‐qPCR

Gene	Forward primer sequence	Reverse primer sequence
TGFβ1	CTGTACATTGACTTCCGCAAG	TGTCCAGGCTCCAAATGTAG
ZEB1	CAGGCAAAGTAAATATCCCTGC	GGTAAAACTGGGGAGTTAGTCA
ZEB2	GAAGACAGAGAGTGGCATGTAT	GTGTGTTCGTATTTATGTCGCA
Snail1	GGCTCCTTCGTCCTTCTCCTCTAC	CCAGGCTGAGGTATTCCTTGTTGC
Snail2	CTGTGACAAGGAATATGTGAGC	CTAATGTGTCCTTGAAGCAACC
Twist1	GTACATCGACTTCCTCTACCAG	CATCCTCCAGACCGAGAAG
Β‐actin	CTACCTCATGAAGATCCTGACC	CACAGCTTCTCTTTGATGTCAC

### 2.13 Statistical analysis

All results were expressed as mean ± standard deviation. One‐way ANOVA was performed between multiple groups using SPSS software (Version 20.0) when homogeneity of variance and normality were met. Otherwise, Dunnett's T3 and nonparametric tests were conducted between multiple groups, respectively. *P* < .05 was considered statistically difference and *P* < .01 was considered significant difference.

## 3 RESULTS

### 3.1 EMT induction by TGFβ1 in MDA‐MB‐231 cells

**Figure 1:**
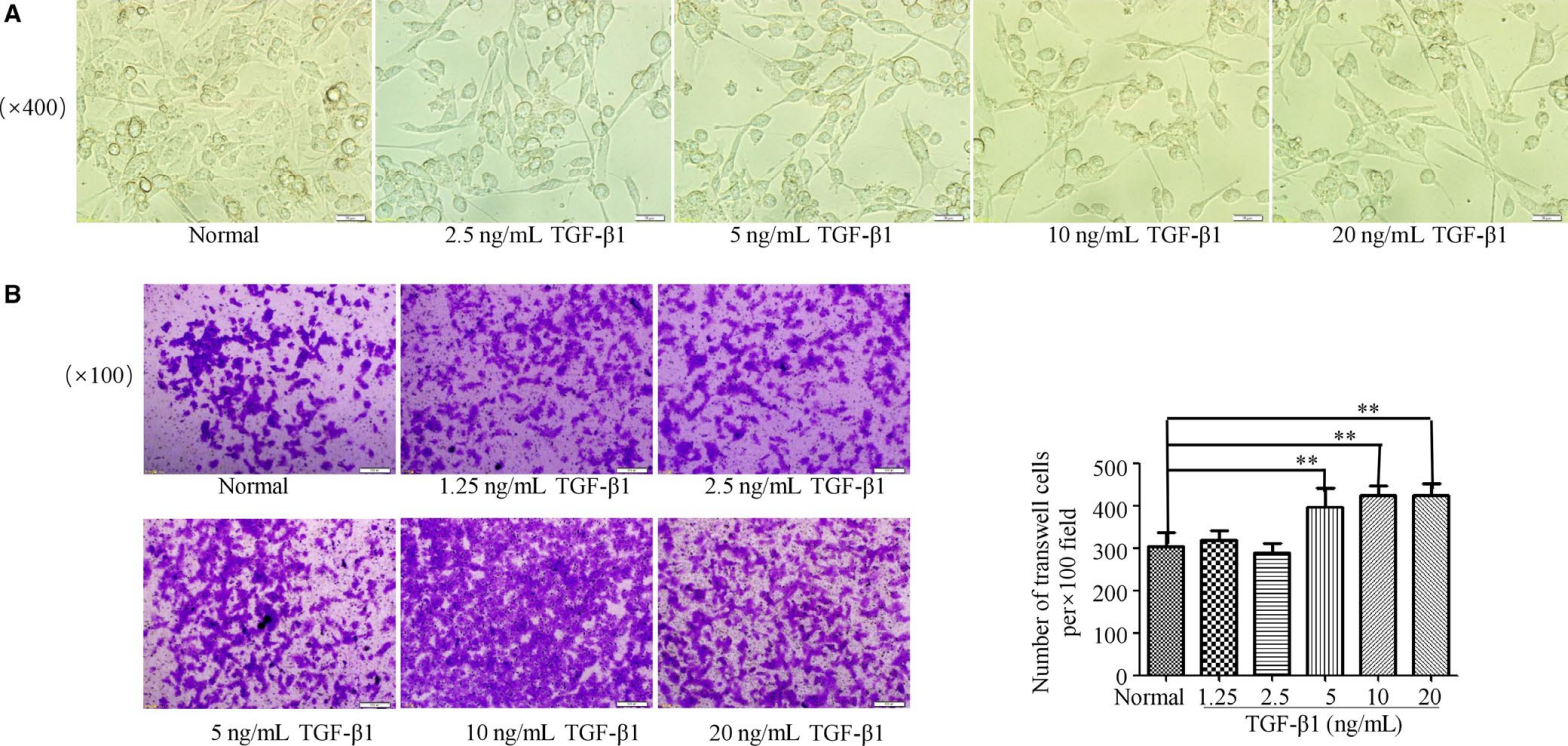
TGFβ1 increased MDA‐MB‐231 cell invasion and affected cancer cell morphology change. A, MDA‐MB‐231 cells were treated with TGFβ1 (0, 2.5, 5, 10, 20 ng/mL) for 48 h, the cell morphology was analyzed by microscopy. The morphology of the cancer cells changed to an elongated spindle shape (original magnification, ×400). B, MDA‐MB‐231 cells were treated with TGFβ1 (0, 1.25, 2.5, 5, 10, 20 ng/mL) for 24 h in transwell, the invasion cells were stained with 0.1% crystal violet. Invasion cell number was significantly increased at 5, 10, 20 ng/mL TGFβ1‐treated cells compared to control (original magnification, ×100). **P < .01

For human triple‐negative breast cancer EMT cells, different concentrations of TGFβ1 (0, 2.5, 5, 10, 20 ng/mL) were administered to MDA‐MB‐231 cells for 48 hours. The results showed that after 48 hours of TGFβ1 induction, MDA‐MB‐231 cells transformed into elongated spindle‐shaped like (Figure 1A). In order to detect the invasive ability of TGFβ1‐treated MDA‐MB‐231 cells (TGFβ1, 0, 1.25, 2.5, 5, 10, 20 ng/mL), transwell invasion assay for 24 hour was performed. The results indicated that the invasive ability of TGFβ1‐treated MDA‐MB‐231 cells was significantly increased, and the invasion ability was more obvious at the concentration of TGFβ1 (5, 10, 20 ng/mL) (*P* < .01) (Figure 1B).

### 3.2 EMT biomarkers and TGF‐β1 protein expression in TGFβ1‐treated MDA‐MB‐231 cells

**Figure 2:**
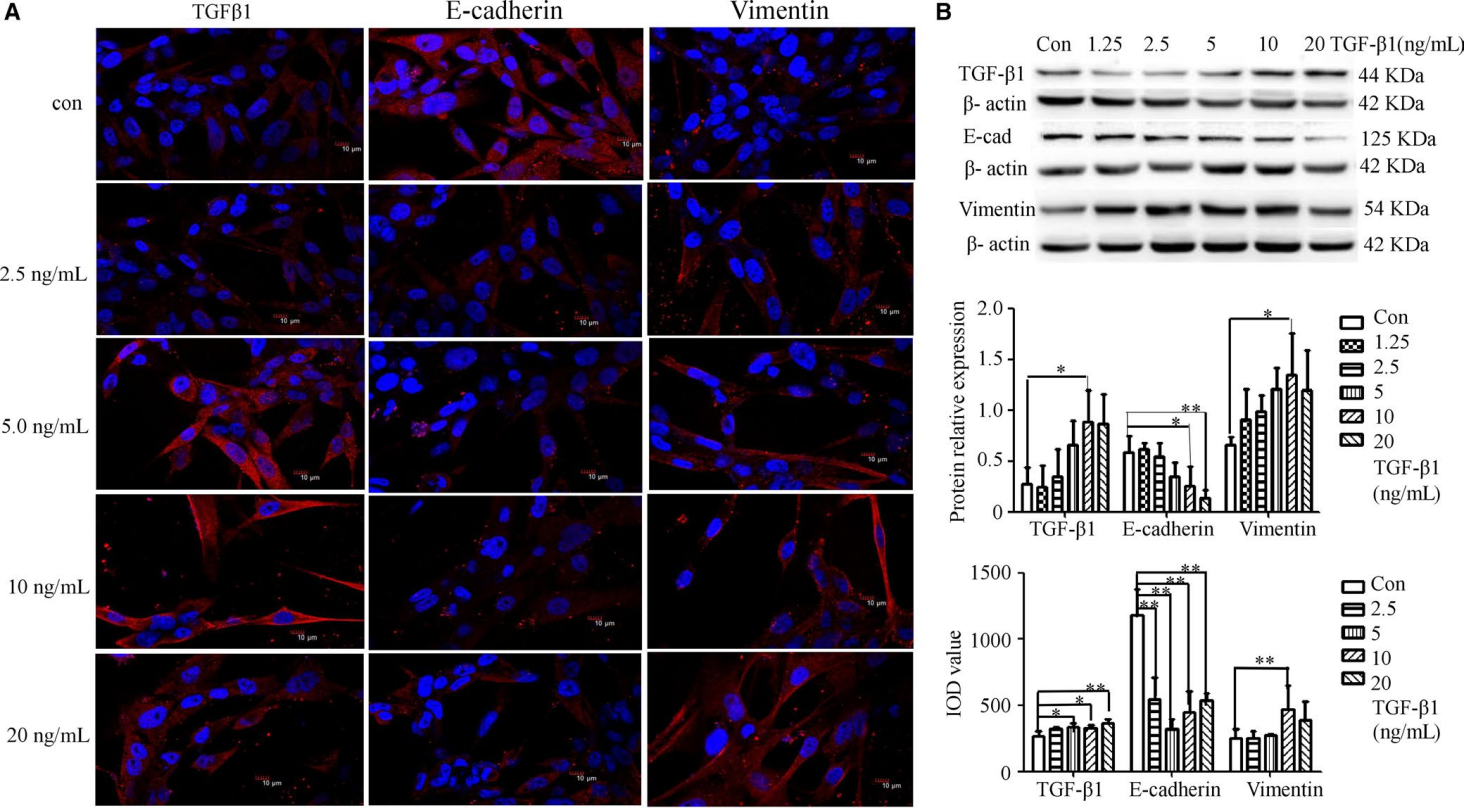
TGFβ1 stimulated MDA‐MB‐231 cell endogenous TGFβ1 and Vimentin expression and inhibited E‐cadherin expression. MDA‐MB‐231 cells were treated with TGFβ1 (0, 1.25, 2.5, 5, 10, 20 ng/mL) for 48 hours. TGFβ1, E‐cadherin, and Vimentin protein level was analyzed by immunofluorescence staining (A) and Western blotting (B). β‐actin was as an inner control. Immunofluorescence staining was observed with the laser confocal scanning microscope (original magnification, ×600). **P < .01, *P < .05, compared with normal control group

In our experiments, immunofluorescence staining showed that E‐cadherin protein was distributed in the nucleus and cytoplasm of MDA‐MB‐231 cells. After 48 hours induction by TGFβ1, Western blotting and immunofluorescence staining results showed that E‐cadherin expression was significantly decreased (*P* < .05, .01). Compared with the normal control group, the protein expression levels of TGFβ1 and Vimentin were significantly increased in TGFβ1‐treated MDA‐MB‐231 cells (*P* < .01, .05) (Figure 2) and interstitial phenotype was enhanced.

### 3.3 Inhibition of Fangjihuangqi Decoction‐medicated serum on proliferation, invasion ability, and morphology in human triple‐negative breast cancer TGFβ1‐treated MDA‐MB‐231 cells

**Figure 3:**
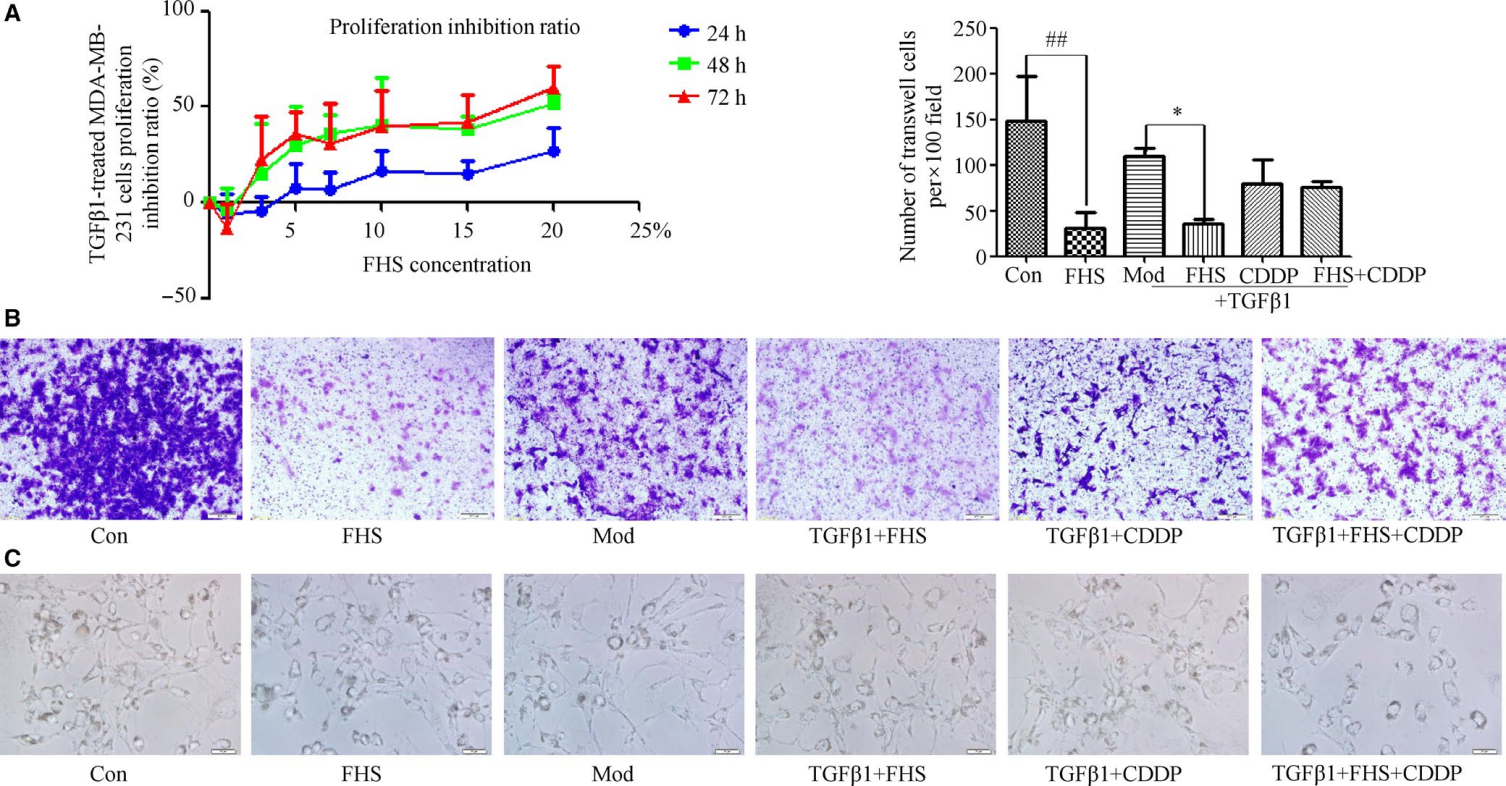
FHS affected both MDA‐MB‐231 cells and TGFβ1‐treated cell morphological change, proliferation, and invasion. A, After FHS treated TGFβ1‐induced MDA‐MB‐231 cells for 24, 48, 72 h, 3‐(4,5‐Dimethylthiazol‐2‐yl)‐2,5‐diphenyltetrazolium bromide assay showed that FHS inhibited TGFβ1‐induced MDA‐MB‐231 cell proliferation. B, Invasive assay after FHS treated 24 h (original magnification, ×100). C, Cell morphological changes of MDA‐MB‐231 cells and TGFβ1‐treated MDA‐MB‐231 cells in different treatment groups (original magnification, ×200). Con group represents the 20% blank‐control serum group, FHS group represents the 20% Fangjihuangqi Decoction‐medicated serum (FHS), Mod group represents TGFβ1 (10 ng/mL) plus 20% blank‐control serum group, FHS + TGFβ1 group represents 20% FHS plus TGFβ1 (10ng/ml), CDDP + TGFβ1 group represents cisplatin (10 μmol/L) plus TGFβ1 (10 ng/mL) and 20% blank‐control serum group, which was as the positive control, and FHS + CDDP + TGFβ1 group represents 20% FHS plus cisplatin (10μmol/L) and TGFβ1 (10ng/ml). *P < .05, compared with TGFβ1‐treated model cells. ##*P* < .01, compared with blank‐control cells

We administered serum‐free medium and different concentrations of FHS (1%, 3%, 5%, 7%, 10%, 15%, 20%). MTT results showed that FHS inhibited the TGFβ1‐treated MDA‐MB‐231 cell proliferation and the IC50 values of 48 hours was 17.77%, with 95% confidence intervals (14.19%‐21.63%) (Figure 3A). We choose 20% as the representative concentration of FHS for the follow‐up experiment. We found that the invasive ability of MDA‐MB‐231 cells was strong and significantly decreased after 24 hours intervention of FHS (*P* < .01). Results also showed that FHS could reduce the invasion ability, reverse EMT cell morphology to make mesenchymal‐epithelial transformation (MET) in TGFβ1‐treated MDA‐MB‐231 cells (*P* < .05) (Figure 3B,C).

### 3.4 Regulation of Fangjihuangqi Decoction on EMT biomarker expression in TGFβ1‐treated MDA‐MB‐231 cells

**Figure 4:**
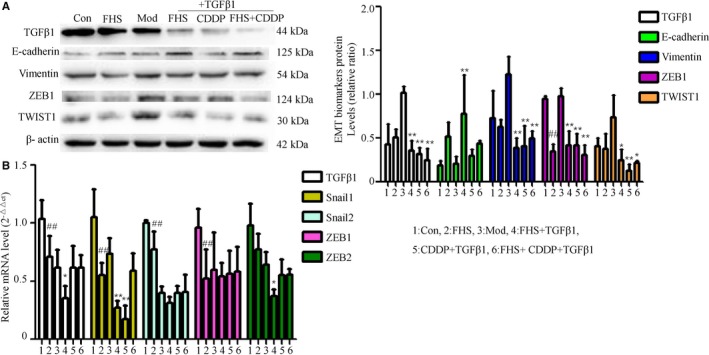
FHS regulated EMT marker expression in MDA‐MB‐231 cells and TGFβ1‐treated cells. The protein expression of TGFβ1, E‐cadherin, Vimentin, ZEB1, and TWIST1 was determined by Western blotting (A). β‐actin was as an inner control. Target protein expression was normalized against β‐actin protein expression. The mRNA expression of TGFβ1, Snail1, Snail2, ZEB1, and ZEB2 was determined by RT‐qPCR (B). The mRNA level of β‐actin was used as an inner control, and gene‐specific mRNA expression was normalized against β‐actin expression. Control group represents the 20% blank‐control serum group, FHS group represents the 20% Fangjihuangqi Decoction‐medicated serum (FHS), Mod group represents TGFβ1 (10 ng/mL) plus 20% blank‐control serum group, FHS + TGFβ1 group represents 20% FHS plus TGFβ1 (10ng/ml), CDDP + TGFβ1 group represents cisplatin (10 μmol/L) plus TGFβ1 (10 ng/mL) and 20% blank‐control serum group, which was as the positive control, and FHS + CDDP + TGFβ1 group represents 20% FHS plus cisplatin (10 μmol/L) and TGFβ1 (10 ng/mL). **P < .01, *P < .05, compared with TGFβ1‐treated model group. ##*P* < .01, compared with blank‐control serum group

Furthermore, we examined the effect of FHS on the protein levels of EMT biomarkers and TGFβ1 in TGFβ1‐treated MDA‐MB‐231 cells. Western blotting showed that compared with EMT model group, FHS could significantly increase E‐cadherin protein expression and decrease the expression of Vimentin, TGFβ1, TWIST1, and ZEB1 proteins (*P* < .01, .05) in TGFβ1‐treated MDA‐MB‐231 cells. Compared with the normal group, FHS could significantly reduce the expression of ZEB1 protein in MDA‐MB‐231 cells (*P* < .01) (Figure 4A). RT‐QPCR results showed that compared with the EMT model group, FHS could significantly reduce the expression of TGFβ1, Snail1, and ZEB2 mRNA in TGFβ1‐treated MDA‐MB‐231 cells. Compared with the normal group, the FHS could significantly reduce Snail1, Snail2, ZEB1, and TGFβ1mRNA expression in MDA‐MB‐231 cells (*P* < .01, .05) (Figure 4B).

### 3.5 Inhibition of Fangjihuangqi Decoction on tumor growth, angiogenesis, and lymphangiogenesis in TNBC xenograft tumor zebrafish

**Figure 5:**
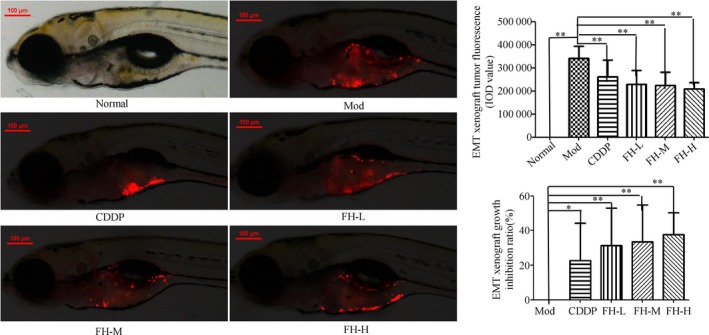
FH inhibited tumor growth in TNBC xenograft zebrafish model. 200 TGFβ1 48 h treated TNBC cells labeled with a red fluorescent dye (CM‐DiI) were injected into 2dpf normal zebrafish yolk sac. After xenograft tumor model was established, the tumor models were treated with FH‐L, FH‐M, FH‐H, CDDP, and control. The fluorescence images were analyzed by a laser confocal scanning microscope. The representative pictures were observed with the laser confocal scanning microscope (original magnification, × 80). The fluorescence IOD was used to evaluate the tumor growth inhibition ratio of Fangjihuangqi Decoction on TNBC xenograft tumor in zebrafish. Image Pro Plus was used to analysis the relative IOD value. Normal represents normal wild‐type AB strain zebrafish, Mod represents the TNBC xenograft tumor model in zebrafish, CDDP represents cisplatin (15 μg/mL), FH‐L represents Fangjihuangqi Decoction (55.5 μg/mL), FH‐M represents Fangjihuangqi Decoction (111 μg/mL), FH‐H represents Fangjihuangqi Decoction (222 μg/mL). **P < .01, *P < .05, compared with model group

Fluorescence microscopy (×100) showed that TNBC xenograft tumor zebrafish model could be established by inoculating TGFβ1‐treated MDA‐MB‐231 cells with CM‐DiI labeled into zebrafish. Compared with the model group, CDDP, FH‐L (55.5 μg/mL), FH‐M (111 μg/mL), and FH‐H (222 μg/mL) groups significantly inhibited the xenograft tumor (*P* < .05, .01) and inhibition ratios were 22.68%, 31.40%, 33.43, and 37.67%, respectively. It indicated that TGFβ1‐treated MDA‐MB‐231 cells had a strong tumor formation ability and FH could significantly inhibit the growth of TNBC xenograft tumor in zebrafish (Figure 5).

**Figure 6:**
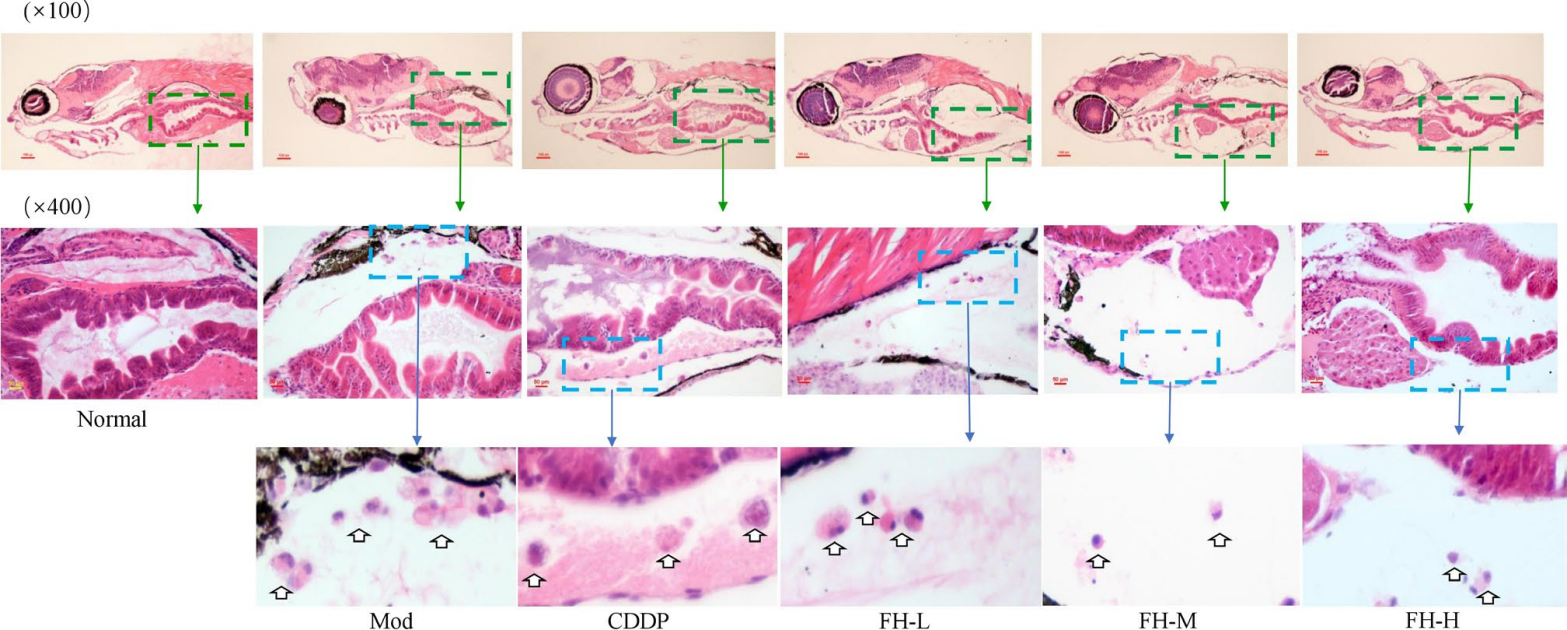
FH inhibited the formation of the histopathological structure of TNBC xenograft zebrafish tumor models. After xenograft tumor model was established, the tumor models were treated with FH‐L, FH‐M, FH‐H, CDDP, and control. The histology was analyzed by microscopy. The green arrow indicates that the tumor cell area observed under ×10 objective lens; the blue arrow indicates that the tumor cell area observed under ×40 objective lens. White hollow arrow indicates the tumor cell. Normal represents normal wild‐type AB strain zebrafish, Mod represents the TNBC xenograft tumor model in zebrafish, CDDP represents cisplatin (15 μg/mL), FH‐L represents Fangjihuangqi Decoction (55.5 μg/mL), FH‐M represents Fangjihuangqi Decoction (111 μg/mL), and FH‐H represents Fangjihuangqi Decoction (222 μg/mL)

From the cell structure analysis, the model group of zebrafish showed more tumor cells in the peritoneal cavity. The tumor cells of the model group had increased pleomorphism, nucleus/cytoplasm ratio, pathological nuclear division, and signet ring cells, which showed characteristics of high invasion, recurrence metastasis tendency, and poor prognosis. In the CDDP group, a small amount of tumor cells was observed in the peritoneal cavity of the TNBC xenograft tumor zebrafish, and the tumor cells increased in volume and the nucleus/cytoplasm ratio. In the FH‐H, FH‐M, and FH‐L groups, the zebrafish abdominal cavity had fewer tumor cells, and the tumor cell nucleus/cytoplasm ratio increased as well as pathological nuclear division. Besides, signet ring cells were not seen except for the FH‐L group. Results also showed that the FH could significantly reduce the number of tumor cells in the abdominal cavity and improve the abnormality morphology of tumor cells (Figure 6).

**Figure 7:**
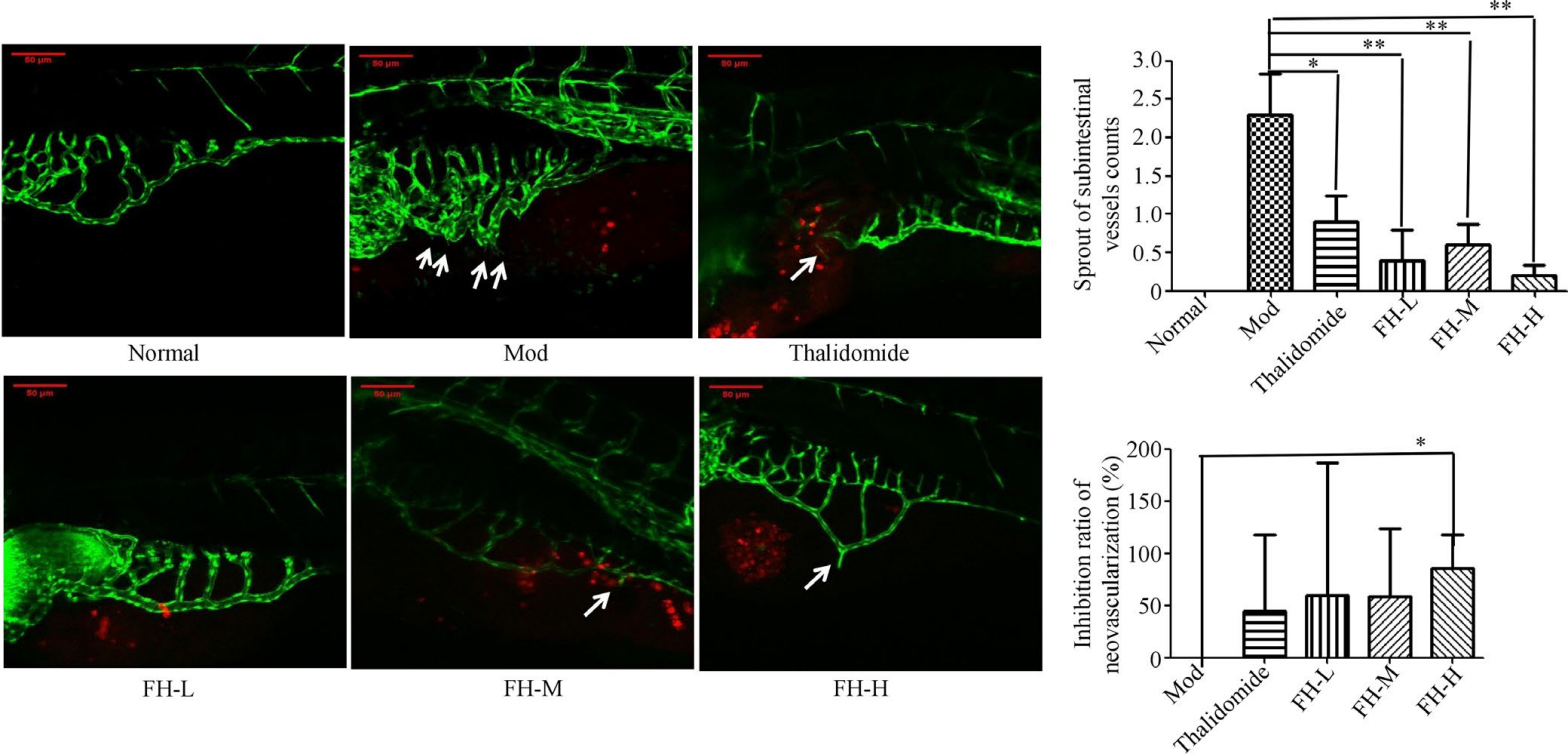
FH decreased tumor angiogenesis in TNBC xenograft zebrafish. 200 TGFβ1 48 h treated TNBC cells labeled with a red fluorescent dye (CM‐DiI) were injected into 2dpf normal transgenic vascular green fluorescent zebrafish yolk sac. After xenograft tumor model was established, the tumor models were treated with FH‐L, FH‐M, FH‐H, CDDP, and control. The fluorescence images were analyzed by a laser confocal scanning microscope. The representative pictures of subintestinal vessel sprout were observed with the laser confocal scanning microscope (original magnification, 200×). White solid arrow indicates sprouts of subintestinal blood vessels. The red is the MDA‐MB‐231 cells of TGFβ1‐treated and labeled with CM‐DiI. The number of buds in the intestine was used to evaluate the inhibition of FH on neovascularization. Normal represents normal transgenic vascular green fluorescent zebrafish, Mod represents the TNBC xenograft tumor model in zebrafish, thalidomide represents thalidomide (219 μg/mL), FH‐L represents Fangjihuangqi Decoction (55.5 μg/mL), FH‐M represents Fangjihuangqi Decoction (111 μg/mL), and FH‐H represents Fangjihuangqi Decoction (222 μg/mL). **P < .01, *P < .05, compared with model group

The intestines blood vessels contour in zebrafish normally take on a basket shape with no extra vascular bud. New blood vessel sprouting is mainly caused by tumor. In the model control group, the intestinal vascular structure was disordered, the diameter was thickened, and the number of buds was 2.30. It was suggested that xenograft tumor zebrafish induced the formation of intestinal neovascularization and the model was established successfully. Compared with the model control group, the intestinal vascular structure and diameter of zebrafish in thalidomide (219 μg/mL)‐positive control group were recovered, the number of buds (0.90) was significantly reduced (*P* < .05), and neovascularization inhibition ratio was 45%, indicating that thalidomide could significantly inhibit intestinal angiogenesis in xenograft tumor zebrafish. In the FH group, compared with the model group, the vasculature was normal, and the diameter was thinner. The sprouting number of FH‐L, FH‐M, and FH‐H groups was 0.40, 0.60, and 0.20, respectively, which was significantly reduced (*P* < .01) than the model control group and the neovascularization inhibition ratios were 60%, 58.33%, and 85.83%, respectively (Figure 7). It was indicated that Fangjihuangqi Decoction could significantly inhibit the tumor neovascularization in TNBC xenograft tumor zebrafish model.

**Figure 8:**
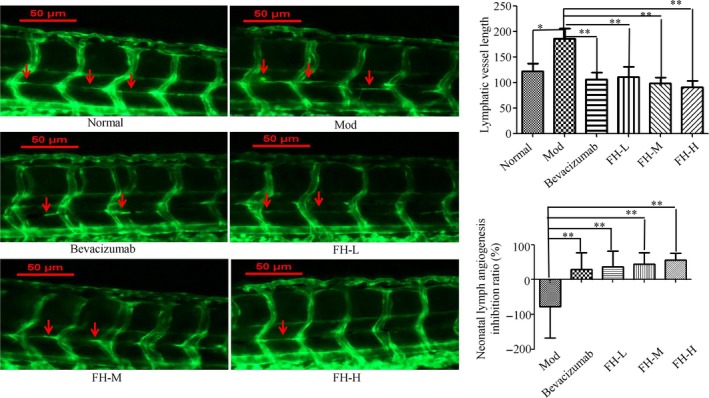
FH affected tumor lymphangiogenesis in TNBC xenograft tumor zebrafish. 200 TGFβ1 48 h treated TNBC cells labeled with a red fluorescent dye (CM‐DiI) were injected into 2dpf normal transgenic vascular green fluorescent zebrafish yolk sac. After xenograft tumor model was established, the tumor models were treated with FH‐L, FH‐M, FH‐H, CDDP, and control. The rhodamine‐labeled dextran (TRITC‐Dextran) intravenously was injected into the zebrafish tumor models for angiography. The lymphatic vessels were observed under a laser confocal microscope (original magnification, 160×). Red arrow indicates lymphatics. The length of lymphatic vessels above the zebrafish cloaca was used to evaluate the inhibition ratio of FH on lymphatic vessels. Normal represents normal transgenic vascular green fluorescent zebrafish, Mod represents the TNBC xenograft tumor model in zebrafish, bevacizumab represents bevacizumab (250 ng/tail), FH‐L represents Fangjihuangqi Decoction (55.5 μg/mL), FH‐M represents Fangjihuangqi Decoction (111 μg/mL), and FH‐H represents Fangjihuangqi Decoction (222 μg/mL). **P < .01, *P < .05, compared with model group

Compared with the normal control group, the length of zebrafish lymphatic vessels in the model group was significantly increased, which indicated that the TNBC xenograft tumor zebrafish induced lymphangiogenesis and was conducive to lymphatic metastasis. Compared with the model group, the length of lymphocytes in zebrafish was significantly lower in the bevacizumab group (*P* < .01), and the lymphangiogenesis inhibition was 29.33%, indicating that bevacizumab significantly inhibited lymphangiogenesis in xenograft tumor zebrafish. Compared with the model group, the length of tumor lymphatic vessels of zebrafish in FH‐L, FH‐M, and FH‐H groups was significantly lower (*P* < .01), and the inhibition ratios were 35.83%, 43.39%, and 55.98%, respectively (Figure 8). It was found that Fangjihuangqi Decoction could significantly inhibit tumor lymphangiogenesis in TNBC xenograft tumor zebrafish.

### 3.6 Regulation of Fangjihuangqi Decoction on EMT biomarkers expression in TNBC xenograft tumor zebrafish

**Figure 9:**
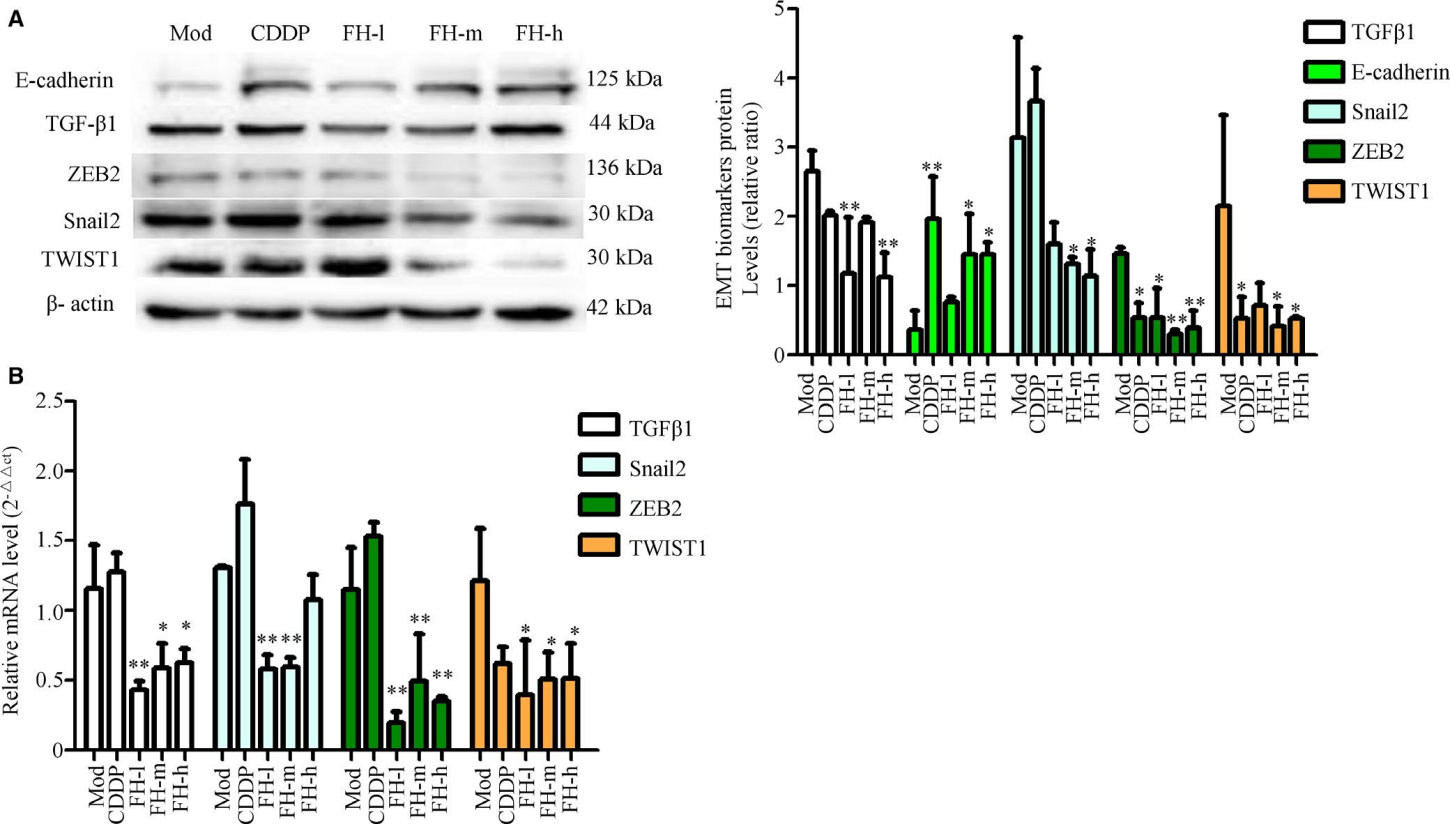
FH moderated EMT marker expression in TNBC xenograft tumor zebrafish. 200 TGFβ1 48 h treated TNBC cells labeled with a red fluorescent dye (CM‐DiI) were injected into 2dpf normal zebrafish yolk sac. After xenograft tumor model was established, the tumor models were treated with FH‐L, FH‐M, FH‐H, CDDP, and control. The protein level of TGFβ1, E‐cadherin, Snail2, ZEB2, and TWIST1 was determined by Western blotting (A). β‐actin was as an inner control. Target protein expression was normalized against β‐actin protein expression. The mRNA expression of TGFβ1, Snail2, ZEB2, and TWIST1 was determined by RT‐qPCR, the mRNA level of β‐actin was used as an inner control, and gene‐specific mRNA expression was normalized against β‐actin expression (B). Normal represents normal wild‐type AB strain zebrafish, Mod represents the TNBC xenograft tumor model in zebrafish, CDDP represents cisplatin (15 μg/mL), FH‐L represents Fangjihuangqi Decoction (55.5 μg/mL), FH‐M represents Fangjihuangqi Decoction (111 μg/mL), and FH‐H represents Fangjihuangqi Decoction (222 μg/mL). **P < .01, *P < .05, compared with model group

**Figure 10:**
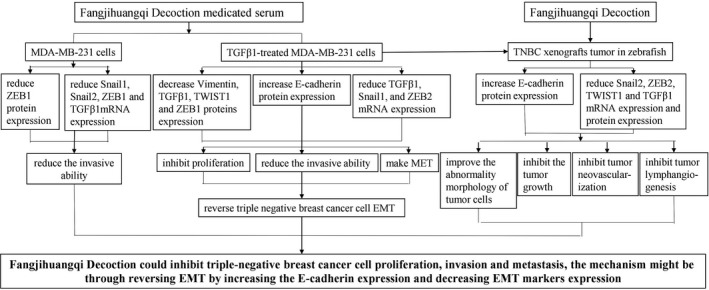
The underlying mechanism chart of Fangjihuangqi Decoction treatment on TNBC. Administration of Fangjihuangqi Decoction prevented the development of TNBC metastasis through reversing EMT by increasing the E‐cadherin expression and decreasing EMT marker expression by in vitro experiment and zebrafish xenograft tumor model

Western blotting showed that compared with the EMT model group, FH could significantly increase E‐cadherin and reduce Snail2, ZEB2, TWIST1, and TGFβ1 protein expression in TNBC xenograft tumor zebrafish (*P* < .01, .05). Cisplatin significantly increased E‐cadherin protein expression and decreased the protein expression of ZEB2 and TWIST1 (*P* < .01, .05) (Figure 9A). RT‐qPCR results showed that, compared with the xenograft tumor zebrafish model group, FH could significantly reduce Snail2, ZEB2, TWIST1, and TGFβ1 mRNA expression (*P* < .01, 0.05) (Figure 9B). The mechanism of Fangjihuangqi Decoction treatment on TNBC is as shown in Figure 10.

## 4 DISCUSSION

EMT is a main step in the initiation of cancer metastasis. Epithelial (E‐cadherin) and mesenchymal (N‐cadherin and Vimentin) biomarkers as well as cell morphological changes are major EMT indicators.^32^ TGF‐β1 is a key player in cancer microenvironment modification and promotion of EMT, which could enhance breast cancer cell tumorigenesis, migration, and invasion.^33^, ^34^ In our study, we demonstrated that compared with control cells, TGFβ1‐treated MDA‐MB‐231 cells exhibited an elongated fibroblast‐like shape. The invasive ability of TGFβ1‐treated MDA‐MB‐231 cells was significantly increased at a concentration of 5, 10, 20 ng/mL TGFβ1. Western blotting and immunofluorescence staining results showed that compared with control cells, expression of Vimentin and TGFβ1 was significantly increased and E‐cadherin expression was significantly decreased. These results indicated that TGFβ1 might activate triple‐negative breast cancer MDA‐MB‐231 cell EMT biomarkers and result in cell morphology changes.

Studies have found that FH contains components of flavonoids, alkaloids, lactones, and saponins, among which fangchinoline, tetrandrine, liquiritigenin, and calycosin have good anti‐inflammatory, antitumor and immune‐enhancing effects.^35^, ^36^ Interestingly, most of the active ingredients of FH are related to breast cancer metastasis. Tetrandrine, a bisbenzylisoquinoline alkaloid isolated from *Radix Stephaniae tetrandrae*, could suppress breast cancer angiogenesis and metastasis.^37^ Our previous report indicated that tetrandrine might induce autophagy in triple‐negative breast cancer MDA‐MB‐231 cells through PI3K/AKT/mTOR signaling pathway.^38^ Astragaloside IV, the major active triterpenoid in *Radix Astragali*, could inhibit breast cancer cell invasion by suppressing Vav3‐mediated Rac1/MAPK signaling.^39^ Astragalus polysaccharide, the active ingredient of *Radix Astragali*, could inhibit the proliferation and induce apoptosis of breast cancer cells by activating macrophages.^40^ Codonolactone is the main bioactive component of *Rhizoma Atractylodis macrocephalae*. Both in vitro and in vivo studies have reported that codonolactone inhibited EMT and breast cancer metastasis by downregulating TGF‐β signaling pathway and Runx2 phosphorylation.^41^ Our experiment results confirmed that FH could prevent tumor metastasis in TNBC xenograft tumor zebrafish and reverse EMT in TGFβ1‐treated MDA‐MB‐231 cells.

Snail1, TWIST1, and ZEB1 are major biomarkers of EMT, which could inhibit E‐cadherin expression and activate genes that contribute to the mesenchymal phenotype.^42^ It was found that TGF‐β1 could reduce the expression of E‐cadherin by inducing ZEB1 and ZEB2 in mouse mammary epithelial cells in vivo.^43^ TGFβ1 also could induce the expression of TWIST1 and Snail1, repressing the expression of E‐cadherin and contributing to cancer‐related EMT in vitro.^44^ Our experiment results demonstrated that compared with EMT model group, FHS could significantly increase the protein expression of E‐cadherin, and reduce Vimentin, TGFβ1, TWIST1, and ZEB1 protein expression in TGFβ1‐treated MDA‐MB‐231 cells. The results of RT‐qPCR showed that FHS could significantly reduce the expression of TGFβ1, Snail1, and ZEB2 mRNA in TGFβ1‐treated MDA‐MB‐231 cells.

Casas et al reported that high expression of TWIST1 and Snail2 was correlated with human breast tumor EMT and metastasis.^45^ Interestingly, Western blotting and RT‐qPCR analysis results showed that the FH could significantly reduce both mRNA and protein level of TGFβ1, ZEB2, TWIST1, and Snail2 in TNBC xenograft tumor zebrafish model. On other hand, the expression of E‐cadherin was increased. Therefore, these results revealed that the FH might significantly inhibit TNBC xenograft tumors and prevent EMT‐induced metastasis in TNBC.

For the metastatic spread of tumor cells, it is very important to grow a vascular network. The processes of new blood vessels and new lymphatics vessels caused by tumors are called tumor angiogenesis and lymphangiogenesis, respectively. Tumor angiogenesis is characterized by their immature characteristics, which weaken their function. Some people have proposed antiangiogenic therapy to correct the structural and functional defects of tumors.^46^ In this study, the gastrointestinal vascular structure of the TNBC xenograft tumor zebrafish model group was disordered, the diameter was thickened, and the number of sprouts was increased. FH significantly decreased the intestinal angiogenesis and vascular structural disturbance in TNBC xenograft tumor model. In addition, the premise of tumor lymphatic metastasis is that tumor cells induce the formation of primitive, neonatal lymphatic vessels, and invade the surrounding lymphatic vessels in the tumor stroma. The proliferation, invasion, and migration of tumor cells can accelerate lymphatic metastasis of tumors.^19^ In our study, we exhibited a strong phenomenon that FH significantly decreased the length of lymphatic vessels and inhibited the tumor lymphangiogenesis in TNBC xenograft tumor models. Therefore, the results revealed that the FH could significantly inhibit tumor neovascularization and lymphangiogenesis to prevent triple‐negative breast cancer metastasis.

However, there are some limitations which should be noted in the current study. It is reported^47^ that 21 chemical components could be detected in the FHS, 11 of which are from the Fangjihuangqi Decoction extract and the other 10 may be metabolites. Ten chemical components were identified, namely Kaempferol‐3‐O‐musk Glycoside, Liquiritin, Calycosin‐7‐O‐D‐glucoside, phellodendrine, Wogonin qisu, Fangchinoline, Isoliquiritin, Tetrandrine, Lico Ricesaponine A3, and Astragaloside II (http://www.cnki.net). Our extraction method of drug‐containing serum in this study was consistent with them. Therefore, we will no longer repeat the contents of the Fangjihuangqi Decoction and drug‐containing serum components.

## 5 CONCLUSIONS

Taken together, we propose a new drug targeting triple‐negative breast cancer. Fangjihuangqi Decoction could inhibit triple‐negative breast cancer growth and metastasis. Although further mechanism studies are needed, this finding provides a new avenue of therapeutic interventions for TNBC patients and precautionary measure for TNBC recurrence and metastasis.

## CONFLICT OF INTEREST

The authors declare that there are no conflict of interest.

## AUTHOR CONTRIBUTIONS

XP conceived and designed the study. YG and YF conducted the study and analyzed the data. YG was a major contributor in drafting the manuscript. All authors read and approved the final manuscript version to be published.

## Data Availability

The datasets used and/or analyzed during the current study are available from the corresponding author on reasonable request.
